# Assessing Digital Maturity of Hospitals: Viewpoint Comparing National Approaches in Five Countries

**DOI:** 10.2196/57858

**Published:** 2025-03-06

**Authors:** Kathrin Cresswell, Franziska Jahn, Line Silsand, Leanna Woods, Tim Postema, Marion Logan, Sevala Malkic, Elske Ammenwerth

**Affiliations:** 1 Usher Institute The University of Edinburgh Edinburgh United Kingdom; 2 Institute of Medical Informatics, Statistics and Epidemiology University of Leipzig Leipzig Germany; 3 Department of Health and Care Science Faculty of Health Sciences UiT The Arctic University of Norway Tromsø Norway; 4 Queensland Digital Health Centre The University of Queensland Brisbane Australia; 5 Amsterdam UMC University of Amsterdam Amsterdam The Netherlands; 6 Digital Leadership and Capabilities, Digital Health and Care Scottish Government Edinburgh United Kingdom; 7 Division Digital Transformation Norwegian Directorate of Health Olso Norway; 8 Institute of Medical Informatics UMIT TIROL, Private University for Health Sciences, Medical Informatics and Technology Hall in Tirol Austria

**Keywords:** digital maturity, hospitals, assessment, decision making, health systems, infancy, Australia, Australian, data collection, data, qualitative thematic analysis, self-reporting, practical feasibility, long-term tracking, local data, monitoring

## Abstract

Digital maturity assessments can inform strategic decision-making. However, national approaches to assessing the digital maturity of health systems are in their infancy, and there is limited insight into the context and processes associated with such assessments. This viewpoint article describes and compares national approaches to assessing the digital maturity of hospitals. We reviewed 5 national approaches to assessing the digital maturity of hospitals in Queensland (Australia), Germany, the Netherlands, Norway, and Scotland, exploring context, drivers, and approaches to measure digital maturity in each country. We observed a common focus on interoperability, and assessment findings were used to shape national digital health strategies. Indicators were broadly aligned, but 4 of 5 countries developed their own tailored indicator sets. Key topic areas across countries included interoperability, capabilities, leadership, governance, and infrastructure. Analysis of indicators was centralized, but data were shared with participating organizations. Only 1 setting conducted an academic evaluation. Major challenges of digital maturity assessment included the high cost and time required for data collection, questions about measurement accuracy, difficulties in consistent long-term tracking of indicators, and potential biases due to self-reporting. We also observed tensions between the practical feasibility of the process with the depth and breadth required by the complexity of the topic and tensions between national and local data needs. There are several key challenges in assessing digital maturity in hospitals nationally that influence the validity and reliability of output. These need to be explicitly acknowledged when making decisions informed by assessments and monitored over time.

## Introduction

Health systems are facing increasing pressure to achieve more with less, and health information technology is widely recognized to tackle existing challenges, although it is still difficult to attribute causal connections between health information technology and health care outcomes [1].

The concept of digital maturity has emerged from the field of organizational studies. It has been conceptualized as an organizational state in which processes are digitalized and accompanied by other organizational transformations [2]. The assumption underlying the concept of digital maturity in health care is that organizations that have higher levels, achieve better processes and outcomes [3,4]. This may include improved quality and operational efficiency through implementing electronic health records (EHRs) and laying the foundation for learning health care systems, artificial intelligence, and precision medicine [5]. We here define an EHR as a digital version of a patient’s paper chart including medical history, treatment, and care over time.

The World Health Organization supports driving the improvement of digital maturity in health systems, aiming toward Universal Health Coverage (UHC) [6]. National approaches to measuring digital maturity have emerged in public health systems relatively recently and are now increasingly applied to inform strategic decision-making [7]. Although some measurements are widely applied (eg, those by the Healthcare Information and Management Systems Society [HIMSS]) [8], there are currently no universally accepted methods for measuring digital maturity in health systems [9]. This may partly be due to the evolving landscape of digital health, where the concept of digital maturity is difficult to define. Some have argued that it needs to be conceptualized as a journey without a definitive endpoint [10], and digital health capability is sometimes used interchangeably with maturity to reflect a continuum of digital growth [11].

To understand how best to assess digital maturity in health systems to facilitate comparisons and sharing of lessons, there is a need to compare initiatives across nations. Although approaches to measuring digital maturity have been analyzed within countries and local organizations [4,12,13], there is currently limited evidence surrounding international comparisons. A nuanced view of the role of digital maturity assessments will help to shape implementation efforts. This should include discussions on the value gained from different digital maturity assessments, focusing on their effectiveness, return on investment, and impact on the quality and safety of care.

We therefore aimed to describe and compare national approaches to assess digital maturity. We hope that this work will contribute to fostering an environment of learning and sharing lessons, which can inform future strategies and international comparisons.

## Methods

Countries included in this review were sampled opportunistically through our existing networks in the International Medical Informatics Association (IMIA) and European Federation of Medical Informatics Evaluation Working Groups on technology assessment and evaluation [14,15]. Snowballing through this network identified additional stakeholders. To be included, we needed to be able to access information on the digital maturity assessment process, and we therefore in some cases worked with policy makers who were involved in the exercise within their countries to gather data. We had to find a balance between including countries that were broadly comparable in relation to existing levels of digital maturity, whilst also being sufficiently different to explore varying approaches. Whilst we acknowledge that including countries at various stages in their digital maturity assessment journey could be valuable, we here focused on countries at similar stages but with different approaches (national, regional), different population sizes, different items with various origins (HIMSS, WHO, expert stakeholder consultations) and variations in EHR systems and coverage. Each of the included countries (Australia [Queensland], Germany, the Netherlands, Norway, and Scotland) has a unique health care system shaped by different policies, regulations, and funding mechanisms. Comparing digital maturity across these varied systems, allowed us to gain insights into how different approaches to health care administration impact the adoption and implementation of digital technologies. Furthermore, cultural attitudes toward technology, patient engagement, and health care delivery differ significantly across these countries and understanding how these cultural factors influence digital maturity is important.

We assigned academic (KC, FJ, LW, TP, and EA) or policy (ML and SM) leads to each of the participating countries, who were responsible for collecting descriptive data. Using the nominal group technique with leads, we cocreated a data collection template table for each country, representing key features and learnings identified through discussions in group meetings. These included a description of the context or setting, rationale and drivers for the digital maturity assessments, tools and methods used to collect digital maturity data, methods used to analyze and disseminate the data, challenges encountered, and lessons learned likely to be relevant to the international community (Multimedia Appendix 1). Missing fields were accepted and leads unable to provide complete datasets were still considered eligible. We then coded items under each table field using NVivo 12 (Lumivero) and extracted areas of convergence and areas of divergence under each of the categories through a qualitative thematic analysis. This involved dragging and dropping information recorded for all countries under each table field as the parent node, examining the coded text from across countries for each of the parent nodes. This led to a consolidation of parent nodes under the overall themes presented in the Results section (context, drivers, methods for data collection and analysis, and challenges encountered). We then, within these wider categories, explored similarities and differences between countries which presented the child nodes. Some of these took a numerical form, in which case we examined ranges, whilst for qualitative data we paid particular attention to summarizing the diverse spectrum of experiences across contexts.

The constructed narrative including key themes (presented below) and implications of our findings were then discussed amongst all members of the team in meetings with country leads.

## Results

A comparative overview of key descriptive data across countries is provided in Table 1. We further elaborate on the emerging themes in the paragraphs below.

**Table 1:** Summary of key descriptive data across countries.

Heading	Queensland (Australia)	Germany	The Netherlands	Norway	Scotland
Health care model	Queensland Health (state government) funds universal free health care	Bismarck model	National Health Insurance	National health and social Insurance	National Health Insurance
Size of country or region (# inhabitants), n	~5 million	~84 million	~18 million	~5.5 million	~5 million
EHR^a^ coverage in hospitals	Varied (some still have paper, but single instance of Cerner across 15 hospitals)	Varied (>10 different vendors)	100% (four core EHR systems)	100%	100%
National or regional approach	Regional	National	National	National	National
Name of EHR	Cerner	Varied	Varied	DIPS, EPIC	TrakCare
Name of digital maturity assessment	HIMSS^b^ Digital Health Indicator	DigitalRadar	Dutch Interoperability Maturity Model	eHealth Monitor	Digital maturity survey
Led by	Queensland Health (government)	Digital Radar Consortium (appointed or commissioned by Ministry of Health)	Ministry of Health, Welfare and Sports	Norwegian Government	Scottish Government and Convention of Scottish Local Authorities
Origin	Bespoke, based on HIMSS	Bespoke based on HIMSS and expert review	Bespoke, based on HIMSS	Bespoke based on criteria recommended in WHO's report on the development of national e-health strategies	Bespoke drawing on methodical review of UK and global models
Number of items, n	121	234	37	30	355

^a^EHR: electronic health record.

^b^HIMSS: Healthcare Information and Management Systems Society.

### Context

The size of populations in participating countries varied significantly from 5 million in Queensland, Norway, and Scotland, to 18 million in the Netherlands, and 84 million in Germany.

None of the included health systems were financed through out-of-pocket models or private insurance-based financing models, and all had UHC. Health systems varied from The Beveridge model, where health care is provided free at the point of care through taxation (Scotland, Norway), The Bismarck model which is characterized by compulsory health insurance related to earnings (Germany, the Netherlands), and mixed models where universal public health insurance funded through tax can be supplemented by private health insurance (Queensland) [16].

All participating countries had a strong governmental focus on interoperability in their national strategies, but some had made more progress than others. This was particularly apparent when looking at regional examples. For instance, Queensland had a state-wide Integrated Electronic Medical Record with Cerner. In other countries, where regions had some autonomy over budgets and procurements, there were a multitude of heterogeneous EHR systems sometimes spanning more than 1 provider organization.

EHR coverage in hospitals was hard to assess, but between 50% and 100% of hospitals used some sort of clinical information system (ie, systems designed to support the operations of specific clinical departments or functions within an organization). Norway, Scotland, and the Netherlands had 100% coverage.

Types of EHR systems varied significantly. Some countries were dominated by 1 vendor (eg, Queensland with Cerner, Scotland with TrakCare), whereas others used a mixture of systems including US-based mega systems (Epic, Cerner) and “home-grown” systems (Norway, Germany, the Netherlands).

Digital maturity assessments were national in all countries except Australia, which assessed the region of Queensland. All were initiated and executed by the Government, except in Germany where the government commissioned a consortium.

Although the focus of this analysis was on digital maturity in hospitals, some digital maturity assessments included several health and care settings, including acute, primary, and social care (Norway, Scotland, and the Netherlands).

All assessments had a longitudinal element, although what indicators were collected at any point in time was determined by local contextual factors such as availability and feasibility. In addition, the list of indicators evolved over time as some were added, and others were removed in line with strategic objectives and feasibility (explicitly mentioned in Germany and Scotland).

### Rationale and Drivers for Digital Maturity Assessments

Across countries, the primary drivers for digital maturity assessments were to inform digital health strategy and priority areas of investment, evaluate national progress, and conduct local benchmarking designed to help participating organizations compare themselves to others. In Scotland, the local use of data to guide and plan digital transformation was emphasized, and the German case also mentioned a desire to compare their digital maturity with international centers of excellence.

Some cases also mentioned the exploitation of data generated and the use of data to drive and provide evidence for progressing interoperability agendas. The Norwegian initiative was for instance designed to provide data for research and analysis in public health, and the Dutch strategy mentions the need to conduct a Societal Cost-Benefit Analysis to define if, when and how mandating digital data exchange can be implemented.

### Tools and Methods Used to Collect Digital Maturity Data

Most countries assessed a combination of common elements of digital maturity, such as strategy, information technology capability, interoperability, governance and management, patient-centered care, skills and behaviors, and data analytics [11]. Which elements were assessed was often based on the context of the country. Queensland was the only region that used a HIMSS Digital Health Indicator [17].

All countries collected data through standardized electronic self-assessment surveys that were completed by local organizations and supported by national stakeholders. Some had, or were in the process of developing, bespoke electronic data collection and analysis platforms. There was significant variation both within and across countries as to which organizational stakeholders completed the survey.

Likert scales were mostly used to measure indicators, whilst some also had some multiple-choice items, percent scales, and the option for limited free text. The number of included items, however, varied significantly across countries and did not always correlate with the number of health and care settings included, the size of populations, or the number of EHR systems. Norway’s national eHealth Monitor included 30 indicators, followed by the Dutch Interoperability Maturity Model with 37 items. Queensland had 121 indicator statements, Germany had 234 items, and Scotland had the largest number with 355 discrete indicators (Scotland also included the widest range of care settings).

Areas of focus also varied significantly depending on how countries divided assessment elements. It was therefore difficult for us to compare where countries placed specific items and where they overlapped. Themes commonly measured across countries included interoperability and information exchange, capabilities and applications, organizational leadership and governance, and technological infrastructures.

Specific areas measured locally in individual countries included person-enabled health and predictive analytics in Queensland; resilience management, clinical, processes, telehealth, and patient participation in Germany; skills and competencies, sustainability, and secondary uses of clinical data in Scotland; laws, regulations, and care processes in the Netherlands; and user satisfaction, and cost-monitoring in Norway.

Most assessments were voluntary but strongly encouraged. None of the countries used penalties. The only compulsory assessment was in Germany, but it was only relevant for hospitals that received national funds to support digital transformation.

### Methods Used to Analyze and Disseminate Digital Maturity Assessment Data

Analysis was mostly centrally coordinated and quantitative in nature. We observed a lack of transparency in collected data and digital maturity calculations across countries.

Queensland used proprietary algorithms provided by HIMSS, which allowed a degree of international benchmarking. These were supplemented by some academically led qualitative analyses. Benchmarking by geographical region or organization was done in some countries (Queensland) but not in others (Scotland). Scotland wanted to gain an understanding of the national landscape to better focus strategic funding. They also wanted the submitting organization to develop improvement plans individually and with their linked organizations, across their local, integrated health and care system. There was an explicit agreement that ranking organizations would potentially hinder data collection and analysis. Some countries (the Netherlands and Scotland) also made (or will make) data available for analysis by local organizations themselves.

Outputs were in most cases disseminated directly to participating organizations (through dashboards and meetings) and externally through open webinars and public reports. They were explicitly designed across countries to inform ongoing eHealth strategy. Queensland was the only setting that had a systematic academic evaluation of the digital maturity assessment and associated findings were published in academic journals and conferences, as well as through HIMSS. Scotland and Queensland established an external steering group comprising of subject matter and policy experts.

### Challenges Encountered and Lessons Learned

Challenges encountered included limitations in the measurements used, such as uncertainties about the validity of constructs for assessing digital maturity. The analysis and data collection process were time-consuming, and indicators evolved, raising questions about the validity of longitudinal elements. Balancing the practical feasibility of the process with the depth and breadth required by the complexity of the topic presented a trade-off. There were also challenges in making international comparisons due to limited relevance in some local contexts. The self-reported nature of measurements introduced likely biases in assessments. For example, individuals reporting may have a specific agenda driving their responses and more knowledge of parts of organizational activities than others. There may also be a degree of “gaming” where respondents either over- or underestimate the levels of digital maturity in their organization to obtain funding. In addition, an overall view of organizational structures and activities is unlikely to be possible from any individual perspective, and efforts invested in reporting activity are likely to be strongly impacted by the existence of local leadership support for the exercise. In addition, there was limited use of qualitative assessment of indicators, with qualitative follow-ups for context and clarification used only to a limited extent.

Some countries recognized that there is a need to address concerns that data might be used for performance management, which may hamper efforts to obtain accurate data.

Costs of digital maturity assessments were significant (although we did not make cost-benefit assessments), including the required resources to collect, complete, analyze, and disseminate the data. In some countries, additional costs included consultancy support for data platforms, data collection, and analysis (Queensland, Scotland, and the Netherlands).

## Discussion

### Principal Findings

Although countries varied significantly in population size, existing EHR systems, settings, and indicators; some common themes emerged. These included a national focus on interoperability, a desire to measure changes over time (and a concurrent difficulty keeping track of progress in line with changing circumstances), a need to inform national digital health strategy, priority areas of investment, local benchmarking and strategy development, at least some alignment of common indicators tailored to local needs, a focus on quantitative measurements, and the self-reported voluntary nature of the exercises.

Common thematic areas across countries included interoperability and information exchange, capabilities and applications, organizational leadership and governance, and technological infrastructures. Analysis was centrally coordinated but data were made largely available to participating organizations. Rigorous independent evaluation was only present in 1 instance.

Challenges encountered included the cost of assessment and time-consuming data collection, potential issues surrounding the validity of measurements, issues with reliably measuring changes over time, and potential biases introduced through self-reporting.

### Strengths and Limitations

Our work is one of the first attempts to describe and compare how different countries measure digital maturity in hospitals. We included only a limited number of countries, chosen through a self-selected sample, which may limit the potential transferability of findings. Our results are also likely to only apply to relatively advanced health systems with high levels of EHR implementations and UHC.

The data for the study were collected directly by the authors in narrative form and from various sources, which is likely to have biased what was included as it was subjectively selected, and individual viewpoints may have influenced the accounts. This method of data collection also meant that the study provided limited detail and did not delve deeply into specific thematic areas and indicators. We observed a significant variation in approaches and the number of indicators used by different countries, which was difficult to account for in the analysis.

As our focus was on providing an overview of approaches, we did not collect specific information on how the data were analyzed within each country and we therefore did not obtain any insights into factors such as regional variations, or differences between digitally mature and less mature sites. This could be a subject of follow-on work.

Despite these limitations, we offer a broad analysis that provides initial insights into the various methods countries have used to assess digital maturity in hospitals and the lessons they have learned from these experiences. Building on this work, there is now an opportunity to explore the identified areas in more detail, potentially leading to a more comprehensive understanding of digital maturity assessments in health and care settings across various health systems.

### Integration of Findings With the Literature

There are many commercially available models for health care providers to assess digital maturity, and there are some general practical considerations that are likely to be relevant across settings [18]. Currently, there are however limited evidence-based methods to assess the quality, use, and efficacy of maturity models and limited guidance on selecting the most appropriate model for any given context [19]. The underlying evidence base for national approaches is even less mature, highlighting the importance of our work. A degree of coordination is likely to be important to streamline efforts and remove duplication, and this will also require consideration surrounding digital infrastructures that can be delivered nationally and those that can remain local [20].

The World Health Organization has recently reviewed tools used to monitor digital health in 8 countries (including Australia and the Netherlands), but these were not specifically explored in relation to digital maturity [9]. Nevertheless, there was a degree of overlap concerning focus. For example, infrastructure and governance as well as specific applications including EHRs, telehealth, and electronic prescribing featured heavily in these assessments, illustrating the interlinked nature of digital health strategy, systems, infrastructures, and digital maturity. The report also points out a lack of indicators for measuring health inequalities and a tendency to prioritize outcomes over processes [9]. This may partly be due to the difficulty to measure processes in health and care digitalization, and a lack of expertise to analyze such data [21].

Approaches to assessing digital maturity have in the past been criticized for relying too heavily on assessing technological capabilities, overshadowing socio-organizational aspects [22]. This imbalance is reflected in the current analysis but there appears to be an increasing recognition of socio-organizational factors such as leadership and governance and consumer or patient-centered care.

The issue surrounding the evolution of intervention maturity over time has already been recognized [23], but how digital maturity assessments can adequately account for this progression is less clear. Changing circumstances are recognized as a significant challenge in measuring the progress of digitalization in health and care, as the use and exploitation of digital systems is often accompanied by wider changes to organizational functioning, in which existing ways of working are transformed [24]. This in turn impacts the way measurements can be compared over time. The challenge is unlikely to be easily resolved and may require defining a limited set of core measurements that will not be significantly affected over time, whilst being flexible in including new emerging indicators.

### Implications for Policy and Practice

There is therefore a trade-off between breadth and depth influencing the scope of assessments. There is also a need to tailor assessments of different settings, as the prevailing “one size fits all” approach whilst providing insights into overall trends, does not help to understand specific organizational circumstances.

The general lack of academic independent validation in digital maturity models and assessments is concerning and raises questions about the reliability and efficacy of existing approaches. There has also been a lack of focus on processes that lead to measured outcomes in assessments. There is further limited evidence on the interrelationships among maturity dimensions meaning that assessments do not provide evidence-based recommendations for improvement.

The role of common indicators across assessments is important. These bring a degree of international comparability, but contextual variations are significant, and digital maturity progress is determined by legal frameworks and financial resources. Scores developed by HIMSS may facilitate a degree of comparability and validation for digital maturity assessments. But standardized international tools may not pay sufficient attention to locally relevant metrics that organizations can use to inform local strategies. Such tools may also present challenges regarding equity and access and there may be commercial conflicts of interest.

Another significant challenge lies in deciding who should be responsible for collecting data, how they represent professional and organizational interests, and how a degree of uniformity across settings may be achieved. This also highlights the necessity of understanding who is best placed to answer specific questions and reiterates the leading organization (Central Government) being explicit in the purpose of the assessment and communicating this repeatedly. A degree of unpredictability regarding how data will eventually be used remains, and so does uncertainty of how individual assessments fit within a wider long-term strategy. Amongst participating organizations, there may be concerns that the data might be used for performance management or resource allocation. Such uses could lead to apprehension and resistance, potentially exacerbating existing inequities. A potential way to address this could include involving organizations more actively in the design of digital maturity assessment tools, perhaps through a process of cocreation. This could then lead to the development of datasets that satisfy both national and local demands, but it is also likely to require compromises on both sides. Policy makers may be faced with different datasets that are not necessarily comparable, but through these will gain insights into local priorities, which can help to facilitate strategic decision making in different, perhaps unanticipated ways.

Going forward, it will be important to assess how these challenges are tackled over time in individual countries, and across the evolving health and care ecosystem including primary and community care. This mapping exercise could also be followed up with more in-depth quantitative or mixed methods work, exploring how digital maturity assessments evolve over time and which aspects are most useful in guiding strategic directions. Incorporating various stakeholder perspectives will be critical, including the voices of health and care providers and patients. We hope that our work provides a steppingstone for achieving this and summarize recommendations for future research to address the challenges identified in Textbox 1.

**Recommendations for future research to address the challenges identified.**

**Recommendations**

Embed real-time independent mixed methods impact, value, and process evaluations in digital maturity assessments from the start to track progress over time and adjust strategy in line with emerging findings. This should focus on:

- Assessing how digital maturity assessments influence decision making locally and nationally.
- Exploring what success means for digital maturity or transformations and develop an internationally agreed framework for measuring associated metrics.
- Assessing which measurements or activities are most impactful, valuable, and cost-effective and why.
- Determining a minimum viable dataset that can be used to make international comparisons.
- Exploring alternatives to self-assessments and clearly distinguish between national and local datasets and their purposes.
- Exploring trajectories and changes over time.
- Exploring incentives and barriers to data collection and analysis amongst various stakeholder groups.

### Conclusions

Digital maturity evaluations are driven by a national or regional impetus to identify both areas of excellence and those requiring investment in digitalization. However, the process of assessing digital maturity is fraught with challenges that require transparency in decision-making and monitoring through evaluation.

Existing work is frequently based on self-reporting, is very costly, lacks academic input, and is often conducted by stakeholders with diverse and at times conflicting agendas. Despite these concerns, having some data, however imperfect, is better than none. This is especially true when it comes to making policy decisions that need substantiation.

National digital maturity assessments need to mature from being a vendor or consultancy-funded project-based exercise toward longitudinal evidence-based and academically led activity characterized by international collaboration to facilitate learning.

## Appendix

Multimedia Appendix 1 Detailed country descriptions.

## Abbreviations

EHR: electronic health record
HIMSS: Healthcare Information and Management Systems Society
IMIA: International Medical Informatics Association
UHC: Universal Health Coverage
