# Supplementary material for: Assessing Digital Maturity of Hospitals: Viewpoint Comparing National Approaches in Five Countries
Source: J Med Internet Res. 2025 Mar 6;27:e57858. doi: 10.2196/57858 (PMC11926443; doi:10.2196/57858)
Supplement: Multimedia Appendix 1 [file jmir_v27i1e57858_app1.docx]

**Table 1.**

| **SCOTLAND** | Kathrin Cresswell ([kathrin.cresswell@ed.ac.uk](mailto:kathrin.cresswell@ed.ac.uk)) Marion Logan (marion.logan@gov.scot) |
| --- | --- |
| **Context** | Conducting regular digital maturity assessments as a commitment within the Scottish Government’s Digital Health and Care Strategy. |
| **Healthcare model (max. 20 words)** | National Health Insurance |
| **Size of country or region (# inhabitants)** | Approximately 5 million |
| **National hospital EHR strategy (in existence since when? Major focus?) (max. 50 words)** | Not to implement EHRs specifically but national move towards an open EHR data platform |
| **EHR coverage in hospitals (# of hospital with EHR) (EHR defined here as a partial or full electronic record of patient core data such as allergies, diagnosis, therapies, medication) (percentage of short verbal description)** | 100% |
| **Types of EHR systems (indicate number of vendors of core EHR systems in hospitals) (max. 10 words)** | 12 of Scotland’s 14 regional NHS boards have TrakCare |
| **National or regional approach to digital maturity (enter “national” or “regional”, if needed max 10 words for explanation)** | National |
| **Name** | Digital maturity survey |
| **Rationale and drivers (why is the benchmarking exercise conducted? What is the primary aim?) (max. 20 words)** | Informing Strategy, Identifying areas for investment, benchmarking organisations, justifying investments |
| **Anticipated outcomes (what are the primary anticipated outcomes of the benchmarking exercise?) (max. 20 words)** | Data to facilitate local and national decision making.  At national and organisational level inform:  Priority setting  Measuring and reporting on progress  Strategic funding plans |
| **Who is leading/driving the benchmarking exercise? (max. 20 words)** | Scottish Government and Convention of Scottish Local Authorities (COSLA) |
| **Origin (how were benchmarking measurement tools developed? Did they draw on existing tools?) (max. 20 words)** | Bespoke model drawing on methodical review of UK and global models |
| **How is the data collected? (what tools are used to collect and analyse benchmarking data? Who collects the data?) (max. 20 words)** | Bespoke data collection platform, bespoke dedicated dashboards for commissioning stakeholders and participating organisations. Analysis by external consultants. Yearly assessment cycle. |
| **Content (how many benchmarking items does the tool contain approximately? Are these free text or numerical?) (max. 50 words)** | 355 discrete indicators (230 Likert / 42 Percent Scale / 83 Other) split by service type where relevant. Indicators grouped into 20 topics each assigned to one of three themes. Staff survey component: 28 Likert indicators. Completion by organisations or jointly by local healthcare and social care systems. |
| **Areas of focus (what overall benchmarking topics/dimensions does the tool address?) (max. 100 words)** | Readiness Theme: Strategic Alignment, Leadership, Resourcing, Governance, Information Governance, Skills and Competences, Climate Emergency and Sustainability  Infrastructure Theme: Enabling Infrastructure, Solutions  Capabilities Theme: Records, Assessments & Plans, Transfers of Care, Orders & Results Management, Medicines Optimisation, Decision Support, Remote and Assistive Care, Digital Channels, Asset and Resource Optimisation, Business and Clinical Intelligence, Standards, Digital Clinical Safety  Staff Survey: Selection from above sections, Benefits Tracking, Roles, Care Settings |
| **Care settings (what care settings does the benchmarking exercise cover?) (max. 20 words)** | General Health and Care  Acute  Primary care  Community  Adult Social care  Children’s Social Care  Mental Health |
| **How is the benchmarking data analysed (nationally? Locally? Qualitatively? Quantitatively?) (max. 20 words)** | Quantitatively/aggregated at national level via dashboard and report; quantitatively/qualitatively at local level via dashboard. No benchmarking by organisation (deliberately) |
| **What are the outputs and how are they communicated? (What is done with the insights obtained through the benchmarking analysis? And who disseminates the benchmarking data to whom and how?) (max. 50 words)** | Outputs by organisation disseminated via self-service analytics dashboard and dedicated tool supporting planning; further, follow-up meetings with ~50% of sample. Benefits tracking tool to be made available once data volume supports it. National outputs disseminated via public report, stakeholder self-service analytics dashboard and bespoke specialist presentations. |
| **Longitudinal element? (Is the benchmarking data collected over longer periods of time? Are different time periods compared?) (max. 20 words)** | Strongly featured, reporting draws comparisons with previous assessment. Dashboards will include longitudinal self-service tools once data volume supports it. |
| **Compulsory? Penalties? (Are there penalties or incentives to complete for participating organisations? Which ones? Is the benchmarking exercise compulsory?) (max. 20 words)** | Organisations are incentivised by benefits extended to them rather than penalised. |
| **Cost/effort involved (what are the cost categories involved in collecting, analysing and disseminating the benchmarking data?) (max. 20 words)** | Developing data collection and dissemination instruments, completing questionnaires, conducting follow-up meetings, analysis and reporting |
| **Issues encountered (what are the main challenges encountered during the benchmarking exercise?) (max. 50 words)** | Trading off practical feasibility of the process with the depth and breadth required by the complexity of the topic, ensuring support for content and method are easy enough to access for all, ensuring that organisations make generous use of provided facilities to collaborate on their assessments. |
| **Evaluation/monitoring (has the benchmarking exercise been evaluated or monitored? If so, by whom?) (max. 20 words)** | Internal and separately external stakeholders steering groups each comprising of subject matter and policy experts. |
| **Lessons (what are lessons learned to date that may be relevant for the international community?) (max. 50 words)** | Involvement of Senior Leadership can vary  Qualitative follow-up is valuable for context and data quality  Important to address concerns that data might be used for performance management  Valuable to identify individual good practice (hence our decision to promote learning via specific development platform. |
| **References (max. 4)** | https://www.digihealthcare.scot/our-work/digital-maturity/  https://www.digihealthcare.scot/digital-maturity-results-published/ |
| **NORWAY** | Line Silsand (line.silsand@uit.no)  Sevala Malkic (sevala.malkic@helsedir.no) |
| **Healthcare model^7^ (max. 20 words)** | National health and social Insurance |
| **Size of country (# inhabitants)** | Approximately 5,5 million |
| **National hospital EHR strategy (in existence since when? Major focus?) (max. 50 words)** | National strategies for interoperability/infrastructure for exchange of information (National Core record, HelseNorge (national platform of information, gives citizens info about their own health/medication, eCommunication with GPs, etc), ePrescription (national service).  Health organisations (hospitals, primary healthcare, GPs etc) make independent decision about which system to procure.  National control, in terms of government budgets but the 4 health regions are largely independent and responsible for hospitals in each region.  Health organisations (hospitals, primary healthcare, GPs etc) make independent decision about which system to procure. |
| **EHR coverage in hospitals (# of hospital with EHR) (EHR defined here as a partial or full electronic record of patient core data such as allergies, diagnosis, therapies, medication) (percentage of short verbal description)** | 100% coverage of EHRs |
| **Types of EHR systems (indicate number of vendors of core EHR systems in hospitals) (max. 10 words)** | **DIPS ASA in 3 of 4 health authorities, EPIC in 1 of 4 health authorities** |
| **National or regional approach to digital maturity (enter “national” or “regional”, if needed max 10 words for explanation)** | National – eHealth Monitor |
| **Name** | National e-health monitor |
| **Rationale and drivers (why is the benchmarking exercise conducted? What is the primary aim?) (max. 20 words)** | National target indicators to monitor effects of national political actions, quality improvement in healthcare services, provide data for research and analysis in public health. |
| **Anticipated outcomes (what are the primary anticipated outcomes of the benchmarking exercise?) (max. 20 words)** | Document whether the objectives of the political guidelines are being realized. |
| **Who is leading/driving the benchmarking exercise? (max. 20 words)** | Norwegian Government |
| **Origin (how were benchmarking measurement tools developed? Did they draw on existing tools?) (max. 20 words)** | The proposed target indicators are based on criteria recommended in WHO's report on the development of national e-health strategies (World Health Organization (2012): National eHealth Strategy Toolkit (who.int)). |
| **How is the data collected? (what tools are used to collect and analyse benchmarking data? Who collects the data?) (max. 20 words)** | Self-assessment, data from national platforms/infrastructures managed by national representatives. |
| **Content (how many benchmarking items does the tool contain approximately? Are these free text or numerical?) (max. 50 words)** | The national e-health monitor consists of 30 different target indicators which are compiled in five strategic objectives corresponding to the national digital eHealth strategy. |
| **Areas of focus (what overall benchmarking topics does the tool address?) (max. 100 words)** | **Overview of indicators and strategic objectives**  The national e-health monitor consists of 30 different target indicators which are compiled in five strategic objectives corresponding to the national digital eHealth strategy.  Reference 2: 24.78% coverage  **Objective 1: Active participation in own and close one’s health**.  Digital health and care services must facilitate that citizens can easily get involved in prevention, treatment, and follow-up of their own and close one’s health. How, when and where health and care services are carried out must to a greater extent be adapted to the citizen's needs. This will contribute to better utilization of competence and capacity.  **Indicators**: National monitoring through HelseNorge (digital platform) **- different indicators by using HelseNorge are;** Number of visits to Helsenorge, Number of logins to Helsenorge – self-service solutions, Most used services on Helsenorge, Citizens visit/use Self-registered diseases: At Helsenorge, citizens can log in to view the different kind content/information and Helsenorge monitors the number of visits to the various pages of HelseNorge.  Monitoring through **Core Record** (National platform) - Citizens' use of privacy settings in the core record, Citizens' own registrations (diseases, allergies) in the core record, Citizens' searches in the core record, clinicians registering critical information/selected information in the core record.  **Citizen survey on e-health** (questionary)  **Nordic health portals** (National Health Portals in the Nordics)  **Patient Travel** represent patients are journeys to and from publicly approved treatment. Patients register the travel via a Self-service solution at HelseNorge.  **Objective 2: Easier working day**.  Healthcare professionals should have access to user-friendly digital tools that provide good decision support and supports administration processes. This will contribute to strengthened patient safety, a reduction in unwanted variation and a more attractive work situation for healthcare professionals.  **Indicator: Healthcare personnel survey on e-health (digital questionary)**; The Directorate of e-Health conduct an annual survey of healthcare professionals' use of, attitudes toward, and satisfaction with digital health services, including both primary and specialist healthcare services  **Objective 3: Health data for renewal and improvement**.  The health and care services, healthcare professionals and the health and care authorities will increasingly make decisions based on data. More data-driven decisions will contribute to better resource utilisation, increased quality, and innovation in the services, as well as better research, health monitoring, emergency preparedness and overall better public health.  **Indicator:**  A national webportal (helsedata.no) aimed to provide faster and more secure access to health data from the country's many health registries and data sources. Monitoring the use of the webportal.  **Objective 4: Available information and strengthened collaboration**.  Digital collaboration and strengthened information management along with increased standardisation will ensure that health information is secure and easily accessible when needed. This will facilitate a more active citizen, better and more effective health care, as well as better data analyses for quality improvement, health monitoring and governance. Objective 5: Cooperation and instruments that strengthen implementation The implementation in the eHealth area will be strengthened through increased cooperation and better use of financial and legal instruments. This will result in coordinated and comprehensive eHealth development that provide sustainable health and care services of good quality.  **Indicators:**  **National platform E-Prescription** enabling national monitoring of different variables; Percentage of e-prescriptions issued in specialist healthcare, E-prescription – issuing and dispensing  **National platform Electronic message exchange (incl. both primary- and specialist health services**.  **Basic data** – which is a technical solution that gives actors in the sector access to relevant registers through information services. Provided by the Norwegian Health Network  HelseNorge – monitor number of “lookup” on patients’ own medication.  **Objective 5: Cooperation and instruments that strengthen implementation**.  The implementation in the eHealth area will be strengthened through increased cooperation and better use of financial and legal instruments. This will result in coordinated and comprehensive eHealth development that provide sustainable health and care services of good quality.  **Indicators:**  National monitoring of **ICT expenses in the health and care sector in terms of,** Distribution of ICT expenses between operating and investment costs in the four different health regions, ICT expenses per employee in the health regions, ICT expenses as a share of total operating costs in the health regions, development in total ICT expenses.  **National platform E-Prescription**; monitoring of Unique prescribing entities using e-prescriptions.  National eHealth strategy(2023-2030) (ehelse.no) |
| **Care settings (what care settings does the benchmarking exercise cover?) (max. 20 words)** | Health and care (hospital, municipalities, GPs and citizens) |
| **How is the benchmarking data analysed (nationally? Locally? Qualitatively? Quantitatively?) (max. 20 words)** | National analysis Quantitatively, focus on figures, |
| **What are the outputs and how are they communicated? (What is done with the insights obtained through the benchmarking analysis? And who disseminates the benchmarking data to whom and how?) (max. 50 words)** | National e-health monitor displaying the results of the data collected through different indicators.  Informing national strategy.  Five strategic objectives represent the focus areas that the sector will collaborate on towards 2030. Each objective has set of key performance indicators which will help to measure achievement of the objective, and the set of the strategic initiatives that presents the most important strategic activities. |
| **Longitudinal element? (Is the benchmarking data collected over longer periods of time? Are different time periods compared?) (max. 20 words)** | Yes.  The target indicators for the five  strategic objectives are determined based on 1) what are relevant indicators and 2) possible to collect at the present time (2023).  There is an addressed need for further development of the target indicators, which starts during 2023. Then, iteratively improvement and adjustment of target indicators will be done, in line with the step-by-step follow-up of the strategy.  Most of the indicators for 2023 are adoption- or perceived performance indicators. When the development of target indicators evolves, the range of result- and effect indicators will increase. An appropriate balance between quantitative and qualitative target indicators is preferred. |
| **Compulsory? Penalties? (Are there penalties or incentives to complete for participating organisations? Which ones? Is the benchmarking exercise compulsory?) (max. 20 words)** | Most of the benchmarking is based on monitoring digital traces in various registries and systems. |
| **Cost/effort involved (what are the cost categories involved in collecting, analysing and disseminating the benchmarking data?) (max. 20 words)** | National government responsible for the monitoring. |
| **Issues encountered (what are the main challenges encountered during the benchmarking exercise?) (max. 50 words)** | See the cell marked GREEN |
| **Evaluation/monitoring (has the benchmarking exercise been evaluated or monitored? If so, by whom?) (max. 20 words)** | See the cell marked GREEN |
| **Lessons (what are lessons learned to date that may be relevant for the international community?) (max. 50 words)** | See the cell marked GREEN |
| **References (max. 4)** | Nasjonal e-helsemonitor - ehelse |
| **AUSTRALIA** | **Leanna (Lee) Woods** |
| **Context** | The state of Queensland in Australia.  In Australia, hospital healthcare is delivered by the states. |
| **Healthcare model^9^ (max. 20 words)** | Queensland Health (state government) funds universal free health care across acute inpatient care; emergency care; mental health and alcohol and other drug services; outpatient care; prevention, primary and community care; ambulance services, and; sub and non-acute care. |
| **Size of country or region (# inhabitants)** | >5million in Queensland |
| **National hospital EHR strategy (in existence since when? Major focus?) (max. 50 words)** | Queensland has a state-wide Integrated Electronic Medical Record (ieMR) with Cerner vendor. |
| **EHR coverage in hospitals (# of hospital with EHR) (EHR defined here as a partial or full electronic record of patient core data such as allergies, diagnosis, therapies, medication) (percentage of short verbal description)** | Cerner ieMR first implemented 2017 at Princess Alexandra Hospital, Brisbane. In 2022, there were 15 hospitals with more going live every year.  At the time of the assessment, 15 individual hospitals across nine health care systems (of total 16 healthcare systems) had the single instance Cerner integrated Electronic Medical Record (EMR) system. The full stack of advanced EMR capability covers the patient journey across various health care sites, and is integrated with computerised provider order entry, ePrescribing, and clinical decision support systems.  The remaining hospitals use paper-based clinical documentation with various levels infrastructure, connectivity, and point of care technologies for integration of business, patient administration, diagnostics and virtual care systems. |
| **Types of EHR systems (indicate number of vendors of core EHR systems in hospitals) (max. 10 words)** | Cerner |
| **National or regional approach to digital maturity (enter “national” or “regional”, if needed max 10 words for explanation)** | “Regional” to reflect the state of Queensland |
| **Name** | HIMSS Digital Health Indicator |
| **Rationale and drivers (why is the benchmarking exercise conducted? What is the primary aim?) (max. 20 words)** | To evaluate the digital health capability in Queensland to inform digital health strategy and investment. |
| **Anticipated outcomes (what are the primary anticipated outcomes of the benchmarking exercise?) (max. 20 words)** | To understand progress made towards the state’s digital health vision. |
| **Who is leading/driving the benchmarking exercise? (max. 20 words)** | Queensland Health (government) |
| **Origin (how were benchmarking measurement tools developed? Did they draw on existing tools?) (max. 20 words)** | The DHI documents the digital capability of health care services (beyond simple assessments of the presence or absence of electronic medical record systems) using outcome driven, specific and balanced measures.  Launched in the global market in 2019, the DHI was developed from a critical analysis of published, peer reviewed digital health literature, and was tested in health care organisations.  *Snowdon A. Digital Health: A Framework for Healthcare Transformation. Illinois, USA: Healthcare Information and Management Systems Society; 2020* |
| **How is the data collected? (what tools are used to collect and analyse benchmarking data? Who collects the data?) (max. 20 words)** | Self-assessment survey administered electronically to each site. Respondents voluntary staff who (1) had an awareness of digital health across the health care system, (2) the ability to network with local workforce to complete the survey accurately, and (3) provide informed consent.  The survey respondents included chief information officers (n=8), chief digital officers (n=2), clinical directors of digital health (n=2), director of information communication technologies (n=2), executive director of medical services (n=1), and chief digital director medical services (n=1). Respondents required at least 2 hours to complete the survey, receiving support and clarification from HIMSS to avoid partial completions. |
| **Content (how many benchmarking items does the tool contain approximately? Are these free text or numerical?) (max. 50 words)** | 121 indicator statements measured on a five-point scale ranging from not enabled to fully enabled covering the dimensions of digital transformation.  Organisational data are collected using 10 demographic questions which do not contribute to the overall DHI score. |
| **Areas of focus (what overall benchmarking topics/dimensions does the tool address?) (max. 100 words)** | The DHI assesses four key dimensions of digital transformation:   1. Interoperability: subdimensions include foundational, structural, semantic and organisational interoperability 2. Person-Enabled Health: subdimentions include personalised care, proactive risk management and predictive population health 3. Predictive Analytics: subdimensions include personalised, predictive and operational analytics 4. Governance and Workforce: subdimensions include data stewardship, policy and decision-making processes, transparency, workforce capacity and competency. |
| **Care settings (what care settings does the benchmarking exercise cover?) (max. 20 words)** | All Queensland Health funded healthcare systems (n=16). Each health care system has variable numbers of health services covering the full spectrum of complexity from quaternary academic hospitals to small rural hospitals. Publicly funded health care systems were assessed in this study with a focus on digital capabilities of the hospitals contained within, and not the private hospitals across the systems. |
| **How is the benchmarking data analysed (nationally? Locally? Qualitatively? Quantitatively?) (max. 20 words)** | The DHI score was calculated for each health care system using pre-built algorithms; proprietary of HIMSS. Through application of this algorithm each DHI dimension (i.e., interoperability; person-enabled health; predictive analytics; and governance and workforce) can be scored from zero to 100. A proprietary algorithm is then applied to calculate a total score (i.e., the total score is not the sum of the dimension scores).  Analysis included:   - Dimension capability – to determine strengths and weaknesses in capability - Regional capability – to examine DHI differences among metro, regional and rural sites - Regional dimension analysis – to examine dimensional differences among metro, regional and rural sites - Digital hospital analysis – to determine differences with EMR sites and non EMR sites - External benchmarking – to benchmark Queensland globally with Oceana (n=7) and North America (n=10). |
| **What are the outputs and how are they communicated? (What is done with the insights obtained through the benchmarking analysis? And who disseminates the benchmarking data to whom and how?) (max. 50 words)** | Academics and HIMSS: Relevant findings are informing the updated state-wide digital health strategic plan.  HIMSS: Co-designed a report with executives from the healthcare systems for their planning.  Academics: various publications, reports, presentations. |
| **Longitudinal element? (Is the benchmarking data collected over longer periods of time? Are different time periods compared?) (max. 20 words)** | First assessment done in 2021. The state government may plan to repeat the process. |
| **Compulsory? Penalties? (Are there Longitudinal element? Which ones? Is the benchmarking exercise compulsory?) (max. 20 words)** | Not compulsory. No incentives. No penalties. There is no national requirement to complete maturity assessments in healthcare.  This was an academic-led grant in partnership with state government and HIMSS. |
| **Cost/effort involved (what are the cost categories involved in collecting, analysing and disseminating the benchmarking data?) (max. 20 words)** | Cost for the DHI.  In-kind cost to healthcare systems to complete the self-assessment survey – approx. 2 hours plus correspondence and education time.  2 year program of research t |
| **Issues encountered (what are the main challenges encountered during the benchmarking exercise?) (max. 50 words)** | 1. The individual indicator statements and algorithm to calculate the DHI scores are proprietary of HIMSS, and the weighting of the dimension scores to generate the total DHI is unknown. We do not believe this will discredit the approach as it still provides a useful benchmark for others employing the DHI. 2. The ability to assess the digital capability at an individual hospital level, longitudinally or objectively was not possible using this study design. 3. The state health system was assessed by aggregating multiple site analyses, and therefore subject to the accuracy of localized assessments. 4. Global comparisons were limited to DHI scores, with no accompanying comparison of health care systems or point-of-care digital health capabilities. |
| **Evaluation/monitoring (has the benchmarking exercise been evaluated or monitored? If so, by whom?) (max. 20 words)** | Academic evaluation |
| **Lessons (what are lessons learned to date that may be relevant for the international community?) (max. 50 words)** | There are many models available commercially for healthcare providers to use to assess their digital health maturity. Currently, there are limited evidence-based methods to assess the quality, utility, and efficacy of maturity models to select the most appropriate model for the given context.  Woods LS, Eden R, Duncan R, Kodiyattu Z, Macklin S, Sullivan C. Which one? A suggested approach for evaluating digital health maturity models. Frontiers in Digital Health. 2022 24 November 2022. doi: 10.3389/fdgth.2022.1045685. |
| **References (max. 4)** | Woods L, Eden R, Pearce A, Wong YCI, Jayan L, Green D, et al. Evaluating Digital Health Capability at Scale Using the Digital Health Indicator. Applied Clinical Informatics. 2022;13(05):991-1001. doi: 10.1055/s-0042-1757554.  Woods L, Dendere R, Eden R, Grantham B, Krivit J, Pearce A, et al. Perceived Impact of Digital Health Maturity on Patient Experience, Population Health, Health Care Costs, and Provider Experience: Mixed Methods Case Study. J Med Internet Res. 2023;25:e45868. PMID: 37463008. doi: 10.2196/45868.  Duncan R, Eden R, Woods L, Wong I, Sullivan C. Synthesizing Dimensions of Digital Maturity in Hospitals: Systematic Review. Journal of Medical Internet Research. 2022;24(3):e32994.  Woods LS, Eden R, Duncan R, Kodiyattu Z, Macklin S, Sullivan C. Which one? A suggested approach for evaluating digital health maturity models. Frontiers in Digital Health. 2022 24 November 2022. doi: 10.3389/fdgth.2022.1045685. |
| **GERMANY** | Elske Ammenwerth, Franziska Jahn |
| **Context** | In line with the Hospital Future Act (2020), the Ministry of Health initiated the evaluation of the digital maturity of hospitals to measure the impact of the Hospital Future Fund. The funding volume of the hospital future fund for digitisation projects in hospitals amounts to 3 billion €. |
| **Healthcare model^[1]^ (max. 20 words)** | Bismarck model |
| **Size of country or region (# inhabitants)** | 84 Mio. |
| **National hospital EHR strategy (in existence since when? Major focus?) (max. 50 words)** | Since 2023, there is a German digitalisation strategy for health care and nursing with a focus on inter-institutional data exchange, interoperability and processes (through German telematics infrastructure), better data for patient care and research as well as beneficial technologies and applications. One specific goal for hospitals: 50 % of all hospitals supported by the Hospital Future Fund should improve  the digital maturity level in at least two categories of the DigitalRadar assessment by the end of 2025. There is no dedicated national EHR strategy for hospitals, as hospital legislation and (partial) funding is the responsibility of the states. Hospitals most likely have their individual EHR strategies. |
| **EHR coverage in hospitals (# of hospital with EHR) (EHR defined here as a partial or full electronic record of patient core data such as allergies, diagnosis, therapies, medication) (percentage of short verbal description)** | Can only be answered indirectly based on the DigitalRadar assessment. The median EMRAM indicator score for the dimension “clinical information system / clinical data repositories (KIS/CDR)” is 90 (min=48, max=100). |
| **Types of EHR systems (indicate number of vendors of core EHR systems in hospitals) (max. 10 words)** | no official numbers available, >10 different vendors [2] |
| **National or regional approach to digital maturity (enter “national” or “regional”, if needed max 10 words for explanation)** | National (different approaches, “DigitalRadar” with largest reach) |
| **Name** | DigitalRadar |
| **Rationale and drivers (why is the benchmarking exercise conducted? What is the primary aim?) (max. 20 words)** | Part of improving the digital infrastructure of German hospitals  Legal basis: Hospital Future Act (Krankenhauszukunftsgesetz). Primary aim: To assess the status of digital maturity in hospitals and measure effects of the Hospital Future Fund by surveys before and after funding (2024) |
| **Anticipated outcomes (what are the primary anticipated outcomes of the benchmarking exercise?) (max. 20 words)** | to evaluate improvements in digital maturity in German hospitals, basis for the development of digital transformation strategies, to compare digital maturity among German hospitals and worldwide (based on EMRAM indicator scores) |
| **Who is leading/driving the benchmarking exercise? (max. 20 words)** | Ministry of Health is the driver and launched a tender to evaluate the digital maturity level and appointed a consortium consisting of academic institutions, companies and HIMSS. |
| **Origin (how were benchmarking measurement tools developed? Did they draw on existing tools?) (max. 20 words)** | Self-Developed by project consortium together with experts  HIMSS EMRAM indicator scores can be derived from DigitalRadar (DigitalRadar comprises around 65% of the EMRAM items). |
| **How is the data collected? (what tools are used to collect and analyse benchmarking data? Who collects the data?) (max. 20 words)** | Self-assessment, collected via standardised online survey by the DigitalRadar consortium |
| **Content (how many benchmarking items does the tool contain approximately? Are these free text or numerical?) (max. 50 words)** | 234 items (mostly single-or multiple-choice, partly free-text options) in seven dimensions (see below). Items cover the funding areas of the Hospital Future Fund as well as the support of clinical, administrative and other data-related processes. |
| **Areas of focus (what overall benchmarking topics/dimensions does the tool address?) (max. 100 words)** | Structures and systems (IT performance indicators, software applications)  Resilience management and performance (IT security, performance and staff satisfaction)  Organisational governance and data management (organisational governance, data management)  Clinical processes (order management, medication management, sample management, documentation of findings, decision support, flexible working, quality and risk management, information access)  Information exchange (information exchange with external actors, information exchange with patients, information exchange of clinical staff, integration of medical devices, interoperability)  Telehealth (emergency room, teleconsiles, teleconsultation, telemedicine networks)  Patient participation (indicators for use, participation possibilities, strategy, access to information) |
| **Care settings (what care settings does the benchmarking exercise cover?) (max. 20 words)** | Hospitals |
| **How is the benchmarking data analysed (nationally? Locally? Qualitatively? Quantitatively?) (max. 20 words)** | Nationally, quantitatively |
| **What are the outputs and how are they communicated? (What is done with the insights obtained through the benchmarking analysis? And who disseminates the benchmarking data to whom and how?) (max. 50 words)** | Public report with anonymised statistics. Key results of the first assessment were also presented in an open webinar. Dashboard with different statistics for the Ministry of Health. Each participating hospital got an individual dashboard. |
| **Longitudinal element? (Is the benchmarking data collected over longer periods of time? Are different time periods compared?) (max. 20 words)** | Yes, measurements were done in 2021 and will be repeated in 2024. |
| **Compulsory? Penalties? (Are there penalties or incentives to complete for participating organisations? Which ones? Is the benchmarking exercise compulsory?) (max. 20 words)** | Compulsory for hospitals funded by the Hospital Future Fund; there are no plans to link the digital radar survey to penalties. |
| **Cost/effort involved (what are the cost categories involved in collecting, analysing and disseminating the benchmarking data?) (max. 20 words)** | One-time funding for the Digital Radar Consortium for the two planned surveys (including collection, analysis and dissemination). Funding sum is not known.  Time efforts for the hospitals: 10 hours (median) for preparation, 6 hours (median) for completing the survey. No fees for participating hospitals |
| **Issues encountered (what are the main challenges encountered during the benchmarking exercise?) (max. 50 words)** | lowest maturity in dimensions  “patient participation”: mean: 3.5 out of 100,  “telehealth”: mean=18.0 out of 100,  “information exchange” mean=25.1 out of 100;  overall DR score mean=33.3 out of 100 |
| **Evaluation/monitoring (has the benchmarking exercise been evaluated or monitored? If so, by whom?) (max. 20 words)** | Yes, by the Institute for Applied Health Services Research (inav GmbH), Health Care Business GmbH (hcb) and Leibniz Institute für Economic Research (RWI) |
| **Lessons (what are lessons learned to date that may be relevant for the international community?) (max. 50 words)** | Comparison of the EMRAM indicator scores of DigitalRadar with EMRAM scores of the USA, Ontario (Canada) and Australia showed that German hospitals do not lag behind the others, i.e., in all these countries the majority of hospitals only reach stage 0; strong relationship between the EMRAM indicator and the DigitalRadar scores; the issues encountered (see above) were addressed by the Hospital Future Fund |
| **References (max. 4)** | [1] Amelung et al. DigitalRadar – Zwischenbericht, 2022, https://www.bundesgesundheitsministerium.de/service/publikationen/details/digitalradar-zwischenbericht.html  [2] Team digitales-gesundheitswesen.de: Welcher KIS-Hersteller stellt wann welche TI-Anwendung bereit? 2022, https://magazin.digitales-gesundheitswesen.de/kis-ti-anwendung/  [3] Bundesministerium für Gesundheit: GEMEINSAM DIGITAL – Digitalisierungsstrategie für das Gesundheitswesen und die Pflege, 2023, https://www.bundesgesundheitsministerium.de/fileadmin/Dateien/3_Downloads/D/Digitalisierungsstrategie/BMG_Broschuere_Digitalisierungsstrategie_bf.pdf |
| **THE NETHERLANDS** | Tim Postema (tim.postema@nictiz.nl ) |
| **Context** | Maturity assessments as part of achieving compliance to the Wegiz (Dutch law for mandatory electronic information exchange in healthcare).  Two tools are available on a national level int his respect:   1. Volwassenheidsscan (VHS) = Dutch Interoperability Maturity Model (DIMM) (more info) 2. Self-assesment: Self-scan.   Both related to specific Data exchanges (use-cases) |
| **Healthcare model^9^ (max. 20 words)** | National Health Insurance |
| **Size of country or region (# inhabitants)** | Approximately 18 million |
| **National hospital EHR strategy (in existence since when? Major focus?) (max. 50 words)** | Strong focus on interoperability of a multitude of heterogeneous EHR systems, both in terms of semantics as on technical standards. Move towards more government coordination since approximately 2018. |
| **EHR coverage in hospitals (# of hospital with EHR) (EHR defined here as a partial or full electronic record of patient core data such as allergies, diagnosis, therapies, medication) (percentage of short verbal description)** | 100% |
| **Types of EHR systems (indicate number of vendors of core EHR systems in hospitals) (max. 10 words)** | With respect to Dutch hospitals (69, including 8 academic hospitals), there are 4 core EHR systems (2021).  (source a.o.  Ziekenhuiszorg \| Aanbod \| Instellingen \| Volksgezondheid en Zorg (vzinfo.nl)  EPD-marktinventarisatie ziekenhuizen 2021: consolidatie EPD-markt zet door \| M&I/Partners (mxi.nl)) |
| **National or regional approach to digital maturity (enter “national” or “regional”, if needed max 10 words for explanation)** | National |
| **Name** | DIMM (Dutch Interoperability Maturity Model): A scan to establish maturity, to plan the roadmap to obligation under the Law on Electronic Exchange of Information in Healthcare. Based on HIMSS models.  Selfscan: A tool for A tool for healthcare providers to help them: check their status and advices how to get ready to adhere to the goals of the roadmap from the DIMM. |
| **Rationale and drivers (why is the benchmarking exercise conducted? What is the primary aim?) (max. 20 words)** | Data to establish the status and gaps within the relevant healthcare providers in relation to the Data Exchange.  Together with the Societal Cost-Benefit Analysis (MKBA in Dutch) to define if/when and how mandating digital Data Exchange can be implemented.  The scan must give insight into what care institutes and caregivers need to do before they can electronically exchange the specific data (gap) |
| **Anticipated outcomes (what are the primary anticipated outcomes of the benchmarking exercise?) (max. 20 words)** | Data to facilitate local and national decision making.  At national and organisational level inform:  Priority setting  Measuring and reporting on progress  Strategic funding plans |
| **Who is leading/driving the benchmarking exercise? (max. 20 words)** | Ministry of Health, welfare and sports (VWS) |
| **Origin (how were benchmarking measurement tools developed? Did they draw on existing tools?) (max. 20 words)** | Self-Developed by project consortium together with experts together with HIMSS.  Starting point was the HIMSS EMRAM/CCMM models. |
| **How is the data collected? (what tools are used to collect and analyse benchmarking data? Who collects the data?) (max. 20 words)** | Self-assessment in an Excel form, which is processed by an algorithm also in Excel. Currently, a webbased platform is developed by the Dutch SDO (Nictiz) to facilitate the assessments. |
| **Content (how many benchmarking items does the tool contain approximately? Are these free text or numerical?) (max. 50 words)** | The questionnaire comprises 37 compliance statements  • Laws and regulation – 3 compliance statements  • Organisation – 9 compliance statements  • Care Processes – 7 compliance statements • Information – 4 compliance statements  • Application – 10 compliance statements  • Infrastructure – 4 compliance statements  • Each compliance statement was rated on a 5-point scale  • Each option of the 5-point scale corresponds to a DIMM Stage (from Stage 0 to Stage 4)  • Each compliance statement has the same weight in both evaluation pilots  • In a workshop setting, initial responses have been discussed with stakeholders from each healthcare provider • The data were analyzed using the DIMM algorithm  • Final achievements have been calculated and findings have been prepared and shared with the client  The questions are ordered to follow the Nictiz 5 layer framework. |
| **Areas of focus (what overall benchmarking topics/dimensions does the tool address?) (max. 100 words)** | Healthcare providers that exchange their data that fall under the scope of the Data Exchange (more info)  The model consists of 5 stages (0-4) and identifies the levels of HIE capabilities from six different perspectives: Laws and Regulation, Organisation, Care Process, Information, Application and IT Infrastructure. |
| **Care settings (what care settings does the benchmarking exercise cover?) (max. 20 words)** | Depending on scope/Data Exchange (use case)  General Health and Care  Acute care  Primary care  Community  Adult Social care  Children’s Social Care  Mental Health |
| **How is the benchmarking data analysed (nationally? Locally? Qualitatively? Quantitatively?) (max. 20 words)** | Self-assessment is analysed by the healthcare institution itself.  The DIMM is used on a national level.  Quantitatively, input for more qualitative research and decisionmaking. |
| **What are the outputs and how are they communicated? (What is done with the insights obtained through the benchmarking analysis? And who disseminates the benchmarking data to whom and how?) (max. 50 words)** | Informing national strategy, feedback to participating organisations for internal purposes  The mentioned webbased tool will support in advice on current and desired maturity levels on each theme.  National outputs disseminated via public report, published on the government websites among others.  Eg:  Volwassenheidsscan (VHS) van de Basisgegevensset Zorg (BgZ) \| Rapport \| Gegevensuitwisseling in de zorg |
| **Longitudinal element? (Is the benchmarking data collected over longer periods of time? Are different time periods compared?) (max. 20 words)** | The same DIMM model is reused over multiple Data Exchanges.  Dashboards will include longitudinal self-service tools once data volume supports it. |
| **Compulsory? Penalties? (Are there penalties or incentives to complete for participating organisations? Which ones? Is the benchmarking exercise compulsory?) (max. 20 words)** | Participation in the maturity scans are voluntary.  The maturity model serves as input for the government to assess the baseline in relation to proposed legislation, the ;self-assessment scan primarily must give insights into what care institutes and caregivers need to do before they can electronically exchange the specific data (gap) |
| **Cost/effort involved (what are the cost categories involved in collecting, analysing and disseminating the benchmarking data?) (max. 20 words)** | Developing data collection and dissemination instruments, completing questionnaires, conducting follow-up meetings, analysis and reporting, consultancy support. |
| **Issues encountered (what are the main challenges encountered during the benchmarking exercise?) (max. 50 words)** | The time consuming effort for healthcare institutions to supply the data.  Representations of the questions to actually assess maturity – it remains a selection of items used. |
| **Evaluation/monitoring (has the benchmarking exercise been evaluated or monitored? If so, by whom?) (max. 20 words)** | Representativity of the results in relation to the questions can be challenged.  The first results are being evaluated with the Ministry of health and Nictiz based on the first surveys. |
| **Lessons (what are lessons learned to date that may be relevant for the international community?) (max. 50 words)** | Confidence in the model and the initial findings appear to align reasonably closely with reality.  The DIMM requires further modification in terms of the question wording and response options. Further tests for ‘logic should take place prior to further roll-out.  There appears to be a clear case for the Dutch MoH to recommend data exchange standards (transport FHIR, DICOM and also content, HL-7) and to monitor the use thereof. Data exchange standards to be mandated over time as well as national clinical coding classification (ICD-10, LOINC, SNOWMED CT) for diagnoses and procedures. The absence of a unified vocabulary leads to miscommunication.  All care providers should be encouraged and incentivised to submit electronic activity data to payers and insurers thereby improving data quality and reimbursement times.  The MoH may consider national indicators to manage system performance and access including staff satisfaction surveys. |
| **References (max. 4)** | Volwassenheidsscan (VHS) = Dutch Interoperability Maturity Model (DIMM) (more info)  Evaluation DIMM: HIMSS PowerPoint Presentation |
